# Making *in vitro* release and formulation data AI-ready: A foundation for streamlined nanomedicine development^☆^

**DOI:** 10.1016/j.ijpx.2025.100393

**Published:** 2025-09-11

**Authors:** Daniel Yanes, Heather Mead, James Mann, Magnus Röding, Vasiliki Paraskevopoulou, Cameron Alexander, Maryam Parhizkar, Jamie Twycross, Mischa Zelzer

**Affiliations:** a School of Pharmacy, University of Nottingham, University Park Campus, Nottingham NG7 2RD, UK; b Global Product Development, Pharmaceutical Technology & Development, Operations, AstraZeneca, Macclesfield SK10 2NA, UK; c Sustainable Innovation & Transformational Excellence, Pharmaceutical Technology & Development, Operations, AstraZeneca, Gothenburg, 43183, Mölndal, Sweden; d Department of Mathematical Sciences, Chalmers University of Technology and University of Gothenburg, 41296 Göteborg, Sweden; e School of Pharmacy, University College London, 29-39 Brunswick Square, London WC1N 1AX, UK; f School of Computer Science, University of Nottingham, Jubilee Campus, Wollaton Road, Nottingham NG8 1BB, UK

**Keywords:** Machine learning, Data science, Artificial intelligence, Pharmaceutics, Nanomedicine, Databases, Drug release

## Abstract

Machine learning and artificial intelligence (AI) is transforming the way pharmaceutical products are developed across drug discovery, process engineering, and pharmaceutics functions. AI for nanomedicine development is enabling faster and more accurate prediction of critical quality attributes (CQAs). However, the full potential of AI is limited by the quality and accessibility of data. Unlike adjacent fields such as the chemical sciences, the pharmaceutics domain lacks curated, open-access databases, particularly for nanomedicines. To address this, here we curate an open-access local database focused on liposomal formulations. The database includes formulation parameters, *in vitro* release (IVR) testing conditions, and digitised drug release data. By evaluating the entries in the database qualitatively and quantitatively, we identified challenges in current data reporting practices. This includes incomplete reporting of formulation and IVR testing conditions, as well as inconsistent quality of drug release plots and their data format. Based on our analysis, we propose a set of data standards and a database structure to support harmonisation for nanomedicine formulation and IVR data. Our open-access database aims to improve data accessibility and transparency to enable the development of robust AI models for IVR and CQA prediction, ultimately streamlining nanomedicine development.

## Highlights

• Open-access database of liposome IVR experiments made available for predictive AI model development.
• Suggested database structure for IVR and formulation data in liposome development, adaptable to other dosage forms.
• Data standards are proposed to ensure IVR and formulation data is AI-ready.

## Graphical Abstract

**Figure:**
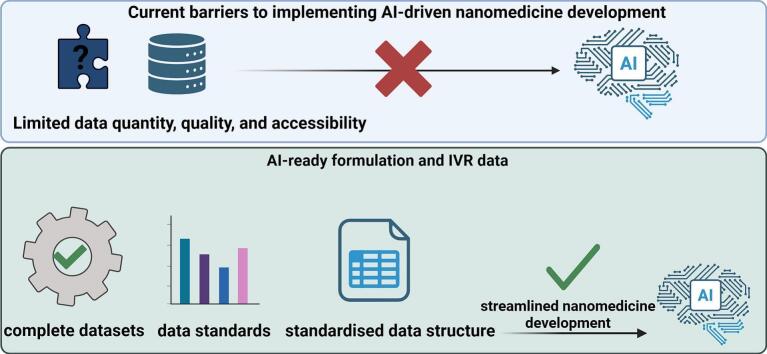

## 1 Introduction

Nanomedicines have increasingly been used to overcome issues with poor aqueous drug solubility, toxicity, and lack of site-targeting after drug administration (Mitchell et al., 2021). Despite their benefits, the regulatory nanomedicine approval process is slow (Đorđević et al., 2022; Jia et al., 2023). This is owed to the complexity of nanomedicine manufacture, non-standardised testing routines, and unpredictable clinical outcomes (Sercombe et al., 2015). In turn, this results in difficulties in translating nanomedicines from bench to market.

During nanomedicine product development, multiple properties are measured to certify the desired quality of the final product (Beg et al., 2019). One critical property is the drug release behaviour. By understanding this kinetic process, the drug release mechanism can be discerned. In addition to assessment of structure-property relationships, the release profile can provide indications of safety, quality, and efficacy of the product. Moreover, *in vitro / in vivo* correlations (IVIVC) can be established to predict *in vivo* performance of a drug in humans (Lu et al., 2011).

**Fig. 1:**
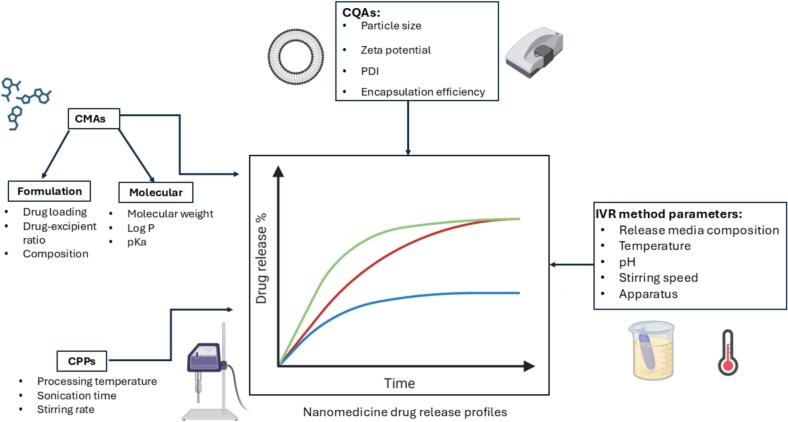
Nanomedicine drug release as a multivariate problem requiring advanced modelling approaches. Parameters influencing release kinetics are grouped into Critical material attributes (CMAs), critical processing parameters (CPPs), critical quality attributes (CQAs) and *in vitro* release *(*IVR*)* method parameters. The listed variables are not exhaustive, and the framework is applicable to multiple nanomedicine classes. Capturing and standardising these parameters is essential for developing robust AI models for predicting drug release.

The drug release kinetics of nanomedicines are governed by numerous factors. Amongst these factors, critical material attributes (CMAs) such as drug and excipient properties and critical process parameters (CPPs) such as processing time and homogeniser speed are closely connected. These factors ultimately lead to critical quality attributes (CQAs) of the formulation such as particle size, zeta potential, and drug loading which impact product performance (Alshaer et al., 2022; Liu et al., 2022). Furthermore, the *in vitro* release (IVR) behaviour of nanomedicines is affected by the choice of release measurement method, media composition, temperature, and pH (Wallace et al., 2012). In Fig. 1, a summary of the variables influencing nanomedicine drug release kinetics is shown. Understanding the individual and combined effects of formulation and process variables on nanomedicine drug release kinetics is challenging, as the problem is multivariate. For instance, particle size is a well-recognised determinant of release rate, with smaller nanoparticles typically releasing their payload more rapidly than larger ones due to their larger specific surface area (Chan et al., 2023). Particle size itself is influenced by formulation component selection, composition, and CPPs, amongst other factors (Maritim et al., 2021; Shaker et al., 2017; Yenduri et al., 2022). Lower molar ratios of cholesterol, have been reported to yield larger particles (Yenduri et al., 2022), which generally release drug more slowly. However, cholesterol concentration also affects membrane fluidity, where higher levels increase bilayer rigidity and reduce permeability of the bilayer (Liu et al., 2000), which can result in slower drug release. A hydrophobic drug such as quinine, which interacts with the non-polar lipid chains, tends to reduce encapsulation efficiency and slows drug release. In this case, increasing the cholesterol content in the particle decreases drug release rates. In contrast, for a hydrophilic drug such as atenolol, the relationship between cholesterol and drug release rate is the reversed. These complexities are further compounded by the effect of the IVR testing conditions on drug release rate. Collectively, these interdependencies highlight the need for machine learning and artificial intelligence (AI) approaches to deconvolute the complex drug release process.

AI methods are increasingly being adopted to accelerate nanomedicine formulation research and development (Bao et al., 2023). AI models can learn to represent complex relationships from data in settings where mechanistic models are difficult to develop or too computationally expensive to use. It has been demonstrated that AI models can be used to accurately predict particle properties (zeta potential or particle size) for varied formulation types such as silica nanofluids (Muneer et al., 2023), amorphous solid dispersions (Schmitt et al., 2022), electro-sprayed polymers (Wang et al., 2022), and liposomes (Han et al., 2023). For IVR predictions, AI has been used to predict release rates for polymeric, long-acting injectables (Bannigan et al., 2023), polysaccharide (Abdalla et al., 2024), and 3D printed tablets (Muñiz Castro et al., 2021). In our recent work, we presented the first application of AI to predict drug release from liposomes (Yanes et al., 2025).

AI approaches require access to large, standardised datasets. In fields adjacent to pharmaceutics, databases such as ChEMBL are available (Gaulton et al., 2012). In pharmaceutics the number of datasets is also growing to expedite the formulation development process *via* AI approaches. These datasets include but are not limited to formulation compositions of self-emulsifying drug delivery systems (Zaslavsky and Allen, 2023) and the drug release behaviour of drug-loaded PLGA microparticles (Bao et al., 2025). Additionally, web-based applications are increasingly used for AI-driven formulation design (Dong et al., 2024; Wang et al., 2025).

At present, in the bio-nano experimental literature, minimum information reporting standards have been suggested to improve reproducibility, facilitate meta analyses, and *in silico* modelling (Faria et al., 2018). However, in the context of nanomedicine AI drug release predictions, data standards are lacking at present and overall adoption of AI in pharmaceutics is limited by the lack of curated databases containing standardised, accessible data for model development (Hickman et al., 2023).

To contribute to tackling the above challenges, this work first proposes a standardised database structure for reporting of liposome IVR data. The database structure is designed to be transferable to other nanomedicine classes such as polymeric, inorganic, and lipid nanoparticle carriers by adapting the formulation and testing parameter fields to system-specific CQAs and CPPs. Liposomes are amongst the most widely researched drug delivery systems (Gu et al., 2023), yet there are no open-access liposome datasets with a focus on IVR data and testing conditions. To fill this gap, we give full access to a comprehensive literature mined database containing 271 distinct IVR profiles, 141 liposome formulations, 22 drugs and extensive details of potential formulation CPPs / CQAs, IVR testing method parameters, and lipid composition features for property prediction. This work expands upon our previously developed ML workflow (Yanes et al., 2025) by offering full open-access to the database used, providing a starting point for formulation, excipient, and IVR method parameter selection. Beyond compiling data, we review the database content to critically assess reporting quality in the literature and propose a set of data standards to establish more consistent and transparent data sharing practices. By making this database open-access, we aim to enhance pharmaceutical industrial-academic data sharing and establish a foundation of data standards for future work on AI-based drug release predictions for other dosage forms.

## 2 Methods

### 2.1 Database construction and data acquisition

The methodology used to construct the database and systematically select literature data is described in the ESI and a summary is provided below.

#### 2.1.1 Database structure

**Fig. 2:**
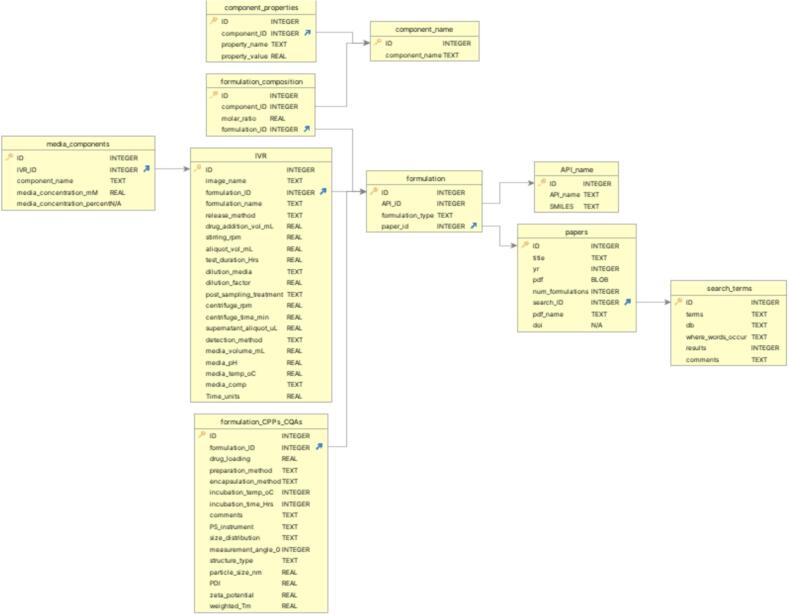
Database structure (schema) for *liposome_IVR.db,* designed to store comprehensive information on IVR tests for liposomal formulations. Each box represents a table in the database. Arrows indicate primary-foreign key relationships between tables. For example process parameters such as preparation method, encapsulation method, incubation temperature, and incubation time and stored in *formulation_CPPs_CQAs* table. While detailed compositional information, including the molar ratio of each formulation component, is recorded in the *formulation_composition* table. The schema enables structured storage and cross-referencing of formulation parameters, process variables, quality attributes, and IVR testing conditions, facilitating efficient data retrieval and modelling. Full table descriptions, field definitions, and an explanation of the schema design are provided in ESI Table S2, ESI section 1.3 and section 2.1.2 in the main manuscript, respectively.

For academic articles that met the criteria above, information relating to the search terms, article, drug used, formulation preparation, characterisation, composition, instrumental details, IVR testing conditions (apparatus, release media composition and conditions, specific details such as amount of drug added) were recorded in a set of 10 related tables. The database was constructed using *SQLite (*v3.43.1*)* within Python (v3.12.1) and tables were defined to manage the one-many relationships encountered in formulation science, such as one formulation being assessed across many different conditions. Full details of all tables, their primary and foreign key which were established to define relationships between the tables, are displayed in the schema derived (Fig. 2, ESI Table S2) and rationale for the schema is given in section 2.1.2. Drug release plots were digitised using *WebPlotDigitizer (*v4.6*)*. Each drug release plot was assigned an integer primary key ID, in the IVR table which was used to name the CSV file which contained the digitised IVR raw data in the form time, release % columns.

#### 2.1.2 Digitalisation of literature data

**Fig. 3:**
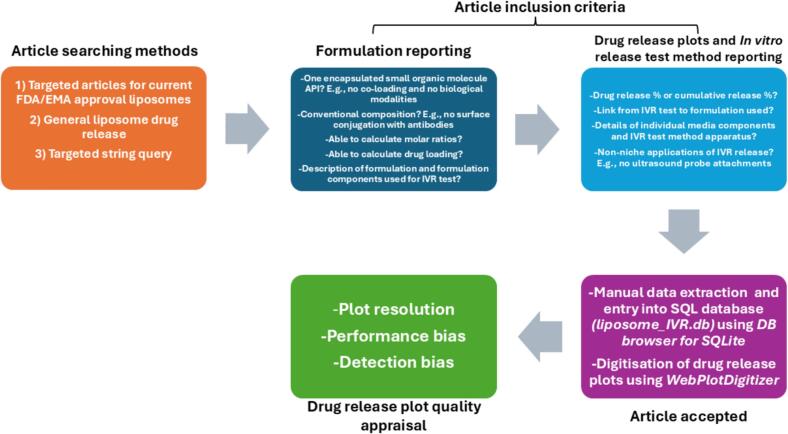
Flowchart outlining the methodology for academic article identification, inclusion and drug release plot quality appraisal. The process starts with three distinct article search strategies, followed by manual evaluation against defined inclusion criteria related to formulation type, composition, *In vitro* release data, and test method reporting. Once accepted, a final drug release plot quality appraisal was performed.

The workflow used for the manual selection of articles and the resultant article inclusion criteria is summarised in Fig. 3. Further details can be found in the ESI, section 1.1.

Data from articles that meet the selection criteria were manually digitised into the database. A manual approach was taken because the way nanomedicine related data *e.g.*, preparation methods, formulation composition, drug loading, analytical techniques, and IVR methodology – are reported in the literature makes automated data extraction very challenging. The development of automated data extraction for nanomedicine data, analogous to the application of large language models (LLMs) in materials science (Dagdelen et al., 2024), could be an attractive avenue for future developments.

To illustrate the digitisation workflow for a single paper, an example is provided in the ESI (section 1.3). Data from each article were entered sequentially across relational tables in a standarised format to reduce redundancy and preserve traceability. Search metadata, including the search terms, database used, and results, were recorded in the *search_terms* table, with each entry assigned a unique ID serving as a foreign key to the papers table containing bibliographic details such as publication year and DOI. APIs mentioned in each paper were stored in the *API_name* table with their chemical identifiers (*e.g.*, SMILES). The *formulation* table linked each API to its parent paper and formulation type, enabling the same formulation to be recorded with different APIs. Components were stored in the *component_name* table, and their proportions were converted or recorded as molar ratios in the *formulation_composition* table, linking component IDs to formulation IDs. This structure allowed a variable number of components to be linked to a single formulation and for commonly used components to be stored efficiently for reuse. Formulation process parameters, characterisation data, and instrumental settings were stored in *formulation_CPPs_CQAs*, enabling multiple characterisation entries for a single formulation for example, preparation methods or measurement angles varied.

IVR experimental details were stored in the *IVR* table, linked to a *formulation_ID*. A single formulation could have multiple IVR entries reflecting different test conditions, such as media composition, temperature, pH, release method, or drug addition volume. The composition of the release media was stored in the *media_components* table, linked back to the *IVR* table, enabling one IVR test to be associated with multiple media components, each with its own concentration or percentage. This also handled cases where the same formulation and IVR method were tested with different media compositions.

The database schema exploited the natural one-to-many relationships in formulation science: one paper could contain multiple formulations, one formulation could have multiple components, CPPs/CQAs, and IVR experiments, and one IVR experiment could involve distinct and multiple media components. This design enabled data traceability, reduced duplication, and supported complex cross-linked queries. An example query, for example, is retrieving all IVR tests for a specific API in a specific media composition containing less than 10 % cholesterol. The database's scalability and standardisation make it suitable for systematic database curation of other nanomedicine systems and for enabling downstream analysis, including AI-based predictions. Adoption of this structure in reporting in the literature could also facilitate future automated LLM-based data extraction.

## 3 Results and discussion

### 3.1 Compiling a comprehensive database of liposome formulations and digitised drug release data

#### 3.1.1 Database construction

To address the lack of a curated database containing liposome formulation characterisation data, IVR testing conditions, and corresponding drug release profiles suitable for AI model development, we systematically mined the literature to compile the first and most comprehensive liposome IVR database. The database captures detailed information on formulation properties, composition, preparation methods, compound identity, formulation characterisation instruments and settings, IVR apparatus and configurations, release media composition, IVR testing conditions, and digitised drug release data (Fig. 2, Table S2).

Our database is designed to serve as a foundation for future AI-driven liposome prediction tasks, including IVR profiles, particle size, and zeta potential. We implemented the database using Structured Query Language (SQL) to accommodate the relational complexity of formulations, where each entry may include multiple excipients with distinct attributes. SQL also enables efficient querying, allowing for streamlined data retrieval and analysis for downstream applications.

A total of 34 academic journals were found using a range of searching methods. During the initial search, it was found that there were differences in the reporting standards of the liposomal IVR testing conditions and apparatus used. Articles were excluded if drug release was reported in unsuitable formats, such as concentration or absorbance readings. In some cases, there were details of preparation of liposomal formulations which were characterised, but an IVR test was not performed. In other scenarios, an IVR test was performed, however there were no details about the preparation of the formulations.

Articles were selected with the aim to develop first a database of liposomal IVR tests. Therefore, all types of liposomes such as small / large unilamellar vesicles and multilamellar vesicles (SUV / LUVs and MLVs) were selected. This meant the time-scale over which tests were conducted varied from seconds, minutes, hours, and days. For articles that met the criteria mentioned (Fig. 3, ESI section 1.1.2), there were also variable reporting standards.

#### 3.1.2 Exploratory database analysis

##### 3.1.2.1 Release methods

**Fig. 4:**
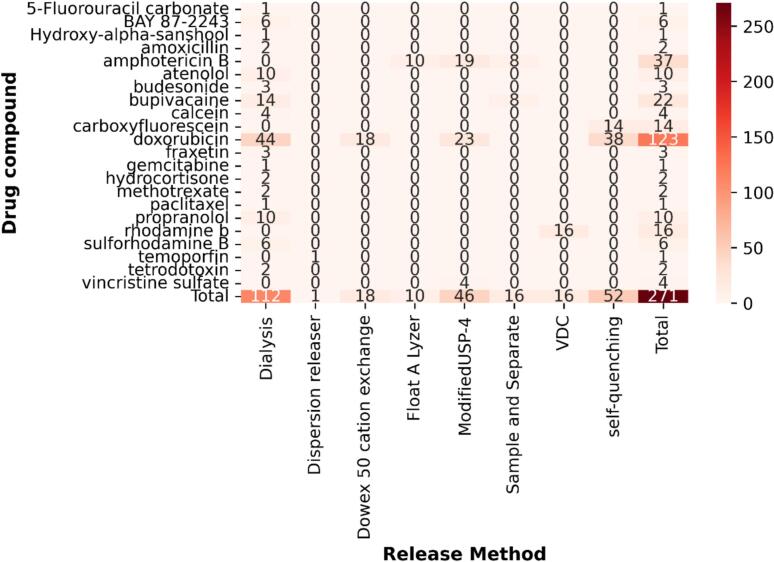
Distribution of drug compounds used with each IVR testing apparatus across all IVR tests in the curated database. Heatmap of drug compound-release method combinations across the dataset, where each cell represents the number of formulations in which a given drug (rows) was tested with a specific release method (columns). Row and column totals summarise the overall frequency of each drug and release method respectively. Differences in apparatus choice can influence measured release profiles as there are no standarised IVR test methods, demonstrating the importance of capturing this data for modelling. Vdc: Vertical diffusion cell.

To assess the diversity of the collated IVR test parameter and formulation characterisation database, exploratory data analysis (EDA) was used. Out of the 271 tests, a total of 22 distinct Active Pharmaceutical Ingredients (APIs) were used, with 45 % of the tests conducted using doxorubicin (Fig. 4). This skew is attributed to the fact that the doxorubicin containing Doxil was the first FDA-approved nanomedicine in 1995, and is considered one of the most effective anticancer drugs to date (Barenholz, 2012). Amphotericin B, a polyene antifungal agent formulated as AmBisome (Stone et al., 2016), accounted for 14 % of the IVR tests.

The heatmap in Fig. 4 highlights the distribution of different release methods used. The dialysis method was the most popular user selected apparatus, accounting for 41 % whilst the dispersion releaser was the least common at 0.4 % of tests (Fig. 4). As both methods are membrane-diffusion techniques, the apparent release data is reported to systematically underestimate the actual drug release rate due to the barrier effect of the dialysis membrane (Yu et al., 2019). It is well documented that there is a lack of standardised protocols in assessing IVR from colloidal drug carriers (Gómez-Lázaro et al., 2024), the diversity in methods used reinforce this. The heatmap highlights sparsity in the curated dataset, revealing underrepresented drug-release method combinations where targeted experimental efforts could improve the accuracy of AI-based drug release predictions.

##### 3.1.2.2 Molecular descriptors

**Fig. 5:**
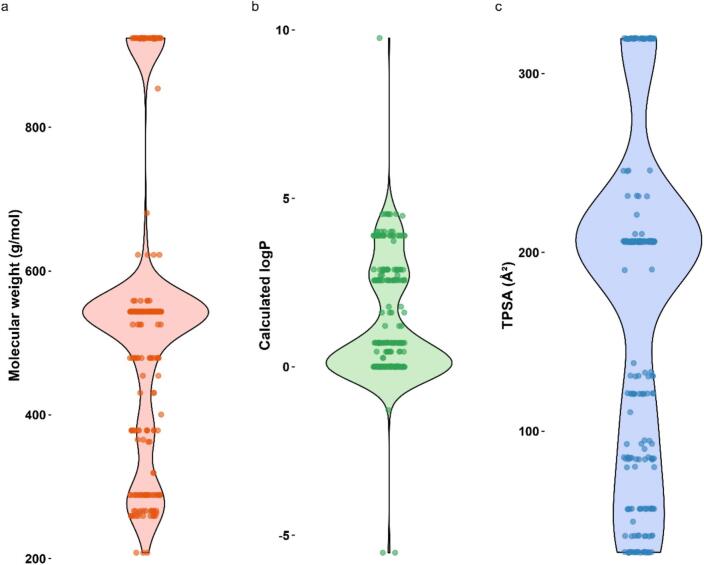
Percent distribution of drug molecular descriptors used in each liposome in each IVR test in the database. Each column shows a violin plot overlaid with individual data points for molecular weight (g/mol) (a), calculated logP (b), and topological polar surface area (TPSA) (c). The violin shape represents the distribution density, while the overlaid points show the actual values for each drug in the dataset.

Each drug in the database was described by several molecular descriptors (Table S13). Fig. 5 highlights the prevalence and distribution of the parameters molecular weight, calculated logP, and topological polar surface (TPSA) in the database, covering ranges of 208–924 g/mol, −5.5–9.8 and 32.3–319.6 Å^2^, respectively. As the drug itself is known to influence the drug release kinetics (Lindner and Hossann, 2010), molecular descriptors were chosen to capture molecular size, polarity, and structural characteristics. The distribution of drug properties used in IVR-tested formulations showed identical groupings because the same drug was used across different lipid compositions or tested under varying IVR conditions and release media (Fig. 5). For effective AI implementation, diversity of training data is required to capture greater information of the system (Gong et al., 2019). The drugs included here covered a parameter space reflective for pharmaceutical products; this does not cover the full, vast range of chemically possible values (Reymond and Awale, 2012). The database, at present, is sufficient for compiling data regarding drug / lipid properties, formulation characterisation, IVR method parameters, and their corresponding digitised drug release profiles.

##### 3.1.2.3 Release media

**Fig. 6:**
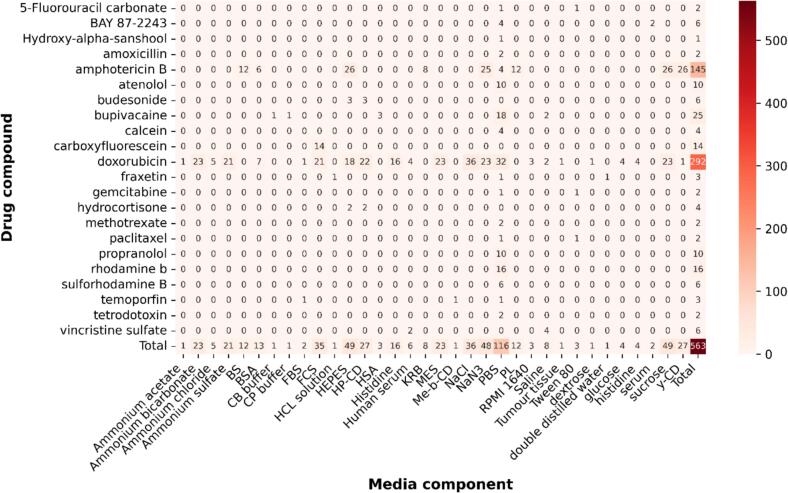
Distribution of drug compounds used with media components in IVR tests across the curated database. Heatmap of drug compound-media component combinations, where each cell represents the number of formulations in which a given drug (rows) was combined with a specific media component (columns). Row and column totals summarise the overall frequency of each drug and media component, respectively. Media components include buffers, surfactants, proteins, and other additives to mimic physiological conditions and influence drug release kinetics. The figure highlights variability in media selection across studies, showing it is important to capture the parameter as a potential input for AI-based drug release predictions.

The release media composition is typically selected to represent physiological conditions, hence phosphate buffered saline (PBS) was the most common buffer selected accounting for 20.6 % of the IVR tests in the database (Fig. 6). Even though PBS was the most commonly used buffer, it is not representative of the complex make up of human blood, which has serum proteins that, upon interaction with liposomes, can destabilise bilayer membranes leading to vesicle disruption (Bonté and Juliano, 1986). Additionally, PBS has a low buffer capacity, which has been reported to lead to pH drift during testing which leads to robustness issues (Mead et al., 2023).

More complex media compositions are employed when developing biorelevant testing conditions to investigate *in vitro* / *in vivo* correlations. For instance, in one of the mined articles, various media components (buffers, synthetic surfactants, and albumin) were screened to assess their impact on the release rate of AmBisome (Díaz de León-Ortega et al., 2021). Amongst these, albumin was identified as the most critical factor influencing release of Amphotericin B.

Overall, liposome drug release is largely governed by thermodynamic properties, such as drug partitioning across the bilayer surface (Jain and Jain, 2016), which in turn are influenced by electrostatic interactions and hence the ionic strength of release media (Boija et al., 2004). As such, the release media composition is a critical parameter that must be carefully selected and controlled when evaluating liposome IVR. Currently, selecting an appropriate release medium largely remains an empirical process that often involves trial-and-error, which is reflected in the variability of media components shown in Fig. 6. Our database aims to address this by allowing users to locally query media compositions, including the range of components employed. This can provide a rational starting point for practitioners selecting a release medium for a new formulation. Furthermore, media composition data may serve as useful feature inputs for future predictive AI models, although the development of media composition featurisation strategies is beyond the scope of this work.

##### 3.1.2.4 Physical liposome properties

**Fig. 7:**
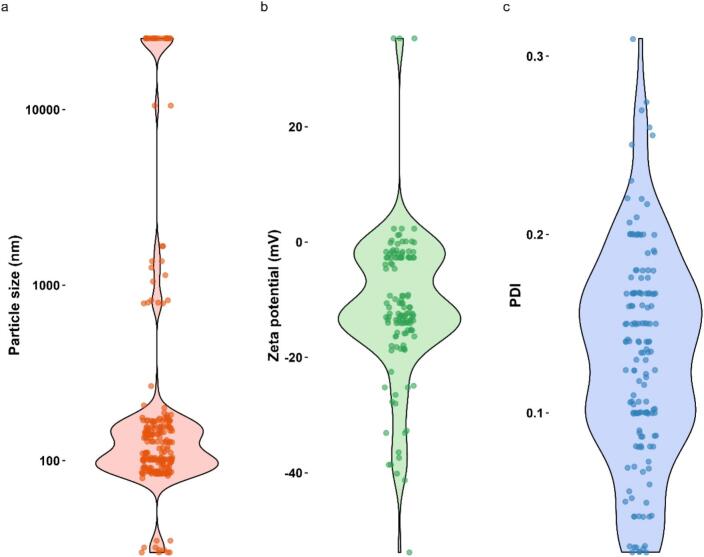
Percent distribution of formulation properties of each liposome used in each IVR test in the database. Each column shows a violin plot overlaid with individual data points for particle size (nm) (a), zeta potential (mV) (b), and polydispersity index (PDI) (c). The violin shape illustrates the distribution density for each property, while the overlaid points represent the measured values for each formulation in the dataset.

The physical properties of liposomes such as particle size, zeta potential, and polydispersity index (PDI) are key parameters known to influence drug release (Alshaer et al., 2022). Particle sizes of the liposomes used in the IVR tests ranged from 30 to 25,500 nm, with a median value of 127 nm (Fig. 7a, b). Particle size of the formulated liposome is optimised during product development. The target size depends on location and type of tissue targeted (Hoshyar et al., 2016), which is why a broad range of particle sizes were measured in the formulations in the database, meaning a range of therapeutic indications were covered. Zeta potential values ranged from −53.8–35.3 mV with a median value of −12.7 mV (Fig. 7c, d). The larger the absolute zeta potential magnitude, the greater the colloidal stability and it is reported that to minimise protein adsorption and improve blood circulation time, liposomes should be close to neutral in terms of surface charge, *i.e.*, within −10 to +10 mV (Smith et al., 2017). The zeta potential values of the formulations in the database fell broadly within this range. The polydispersity index (PDI) of liposomal formulations in the database ranged from 0.022 to 0.31, with a median value of 0.135 (Fig. 7e, f). PDI values <0.3 represent homogenous and well-dispersed systems (Amasya et al., 2016), indicating that the database entries fall within this category.

##### 3.1.2.5 IVR testing conditions

**Fig. 8:**
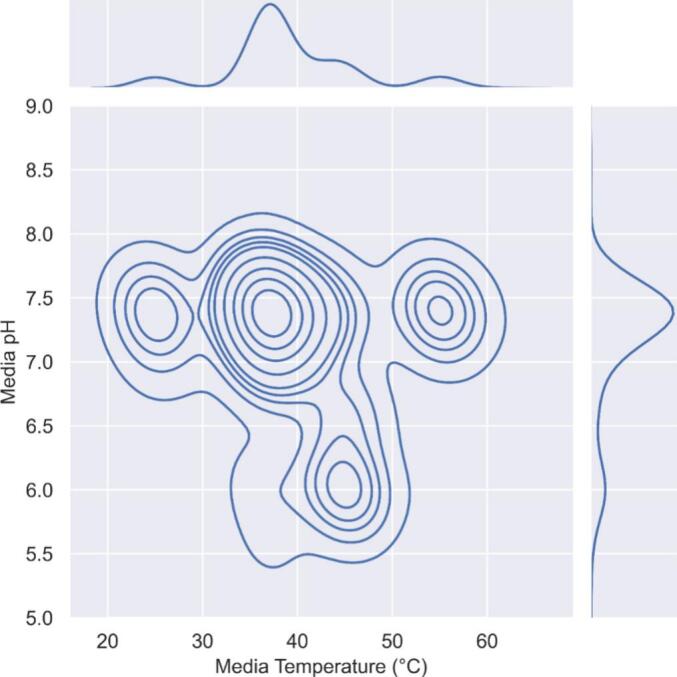
Bivariate distribution of IVR testing parameter space across the curated database, shown as contour plots representing regions of high and lower density. Each contour reflects the frequency with which specific combinations of media temperature and pH occur. More tightly packed contours indicate parameter ranges that are more frequently reported, while wider contours show underexplored regions of the testing parameter space. This visualisation helps reveal trends in the reported IVR test conditions and potential gaps to populate the database.

Typically, IVR tests are conducted at 37 °C and a pH of 7.4 to mimic physiological conditions. This is shown in the bivariate distribution plot of the IVR tests mined from the literature, where a high-density region is identified at these ‘standard’ conditions (Fig. 8). The variance in media temperature (20–60 °C) can be attributed to distinct types of IVR tests such as extended or accelerated release that are employed. The pH values observed ranged from 5.5 to 8.0. The IVR tests conducted with lower pH values contained doxorubicin, which is weakly basic due to the primary amine located on the 3 position on the pyranozide moiety (Teranishi et al., 2016). At lower pH, higher faster release kinetics occur due to conversion into the cationic hydrophilic form. The rationale for the selection of lower pH values can be attributed to either accelerated testing strategies (Shibata et al., 2015) or representative of tumour physiology (Silverman and Barenholz, 2015).

The choice of IVR testing conditions depends on the test's objective: whether to demonstrate drug release behaviour, assess biorelevance, or perform accelerated testing. However, our database reveals significant variability in release media components, IVR conditions, and apparatus selection, highlighting the trial-and-error nature of this process. For example, developing an accelerated IVR test for a new liposome product often requires extensive experimentation, as optimal parameters for one formulation may not apply to another with different drug or lipid compositions. Overall the plots in the previous sections identify clear gaps in the database that provide a basis for decision making for future experimental design plans for experimentalists to rationally expand the dataset.

Our database provides a valuable starting point for querying appropriate IVR conditions for new formulations. AI model development demands standardised data (Suriyaamporn et al., 2024). Therefore, we assessed the completeness of potential feature inputs for AI-driven IVR prediction models.

### 3.2 Incomplete reporting of formulation and testing conditions restricts feature input availability for AI-driven IVR prediction models

**Fig. 9:**
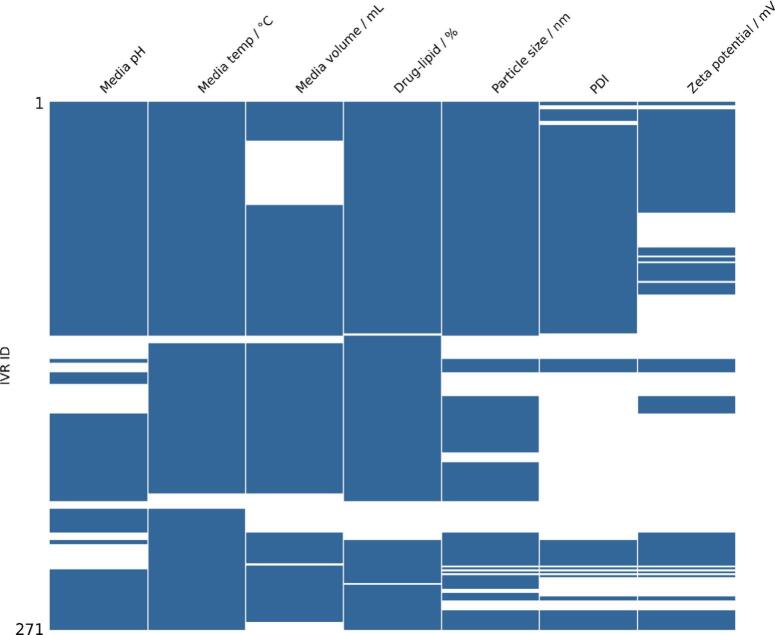
Matrix of potential feature inputs for AI-based drug release prediction across the curated database containing a total of 271 entries. Each row represent a unique IVR test identified by its IVR ID, while each column corresponds to a specific feature digitised from the articles. White spaces indicate missing values for that feature in the corresponding IVR test. This visualisation highlights data completeness and gaps across the joined dataset that can be used for model development.

Robust AI models rely on complete datasets, however incomplete reporting in formulation and IVR testing details significantly limits the data quantity available for model development, reducing predictive accuracy. After our database curation, it was found that with respect to formulation characterisation reporting, specific formulation CQAs for zeta potential, PDI, and particle size were missing in 54 %, 45 %, and 20 % of the IVR entries, respectively. As for IVR test reporting, the release medium volume, pH, and temperature were missing in 23 %, 20 % and 4 % entries, respectively (Table 1). Full reporting of IVR testing conditions and formulation characterisation of the formulation used for testing is therefore required to ensure databases are complete and extracted data subsets are larger. Additionally, data scarcity of the joined dataset highlighted in Fig. 9 is a common issue faced in AI-driven analysis, where there are inconsistencies in reporting between different articles. To overcome these two issues, here, a database structure is suggested for reporting nanomedicine IVR tests and characterisation of the respective formulation (Fig. 2).

**Table 1:** Percent of missing values for potential feature inputs for AI-based drug release prediction across the curated database. Each feature corresponds to a CQA or IVR method parameter that could be used for AI model development. The table highlights data sparsity issues that may impact downstream predictive modelling.

Potential feature input	% missing
Zeta potential / mV	54
PDI	45
Media volume / mL	23
Particle size / nm	20
Media pH	20
Drug-lipid / %	8
Media temperature / °C	4

By implementing the proposed database structure (Fig. 2), the way in which liposomal IVR tests are reported would be standardised to include full details of formulation characterisation, IVR testing conditions, and reporting of instrumental settings to facilitate implementation of future AI analysis and provide improved traceability and reproducibility. Overall, the open-access local database presented is hoped to encourage and foster a collaborative approach between academia and industry to work towards a common goal of saving time/resources in the research and development process of liposome development. In the future, it is anticipated such a database could be hosted on a web server where scientists consistently upload nanomedicine IVR and characterisation data in a structured format specified by the database schema (Fig. 2) which would feed into AI pipelines. To develop AI-prediction models requires a target output to be linked to the feature inputs, in the case of drug release prediction for nanomedicines, this requires access to high quality digitised drug release data.

### 3.3 Inconsistent drug release plot quality and missing raw data limit feature output availability for AI-driven IVR prediction models

**Fig. 10:**
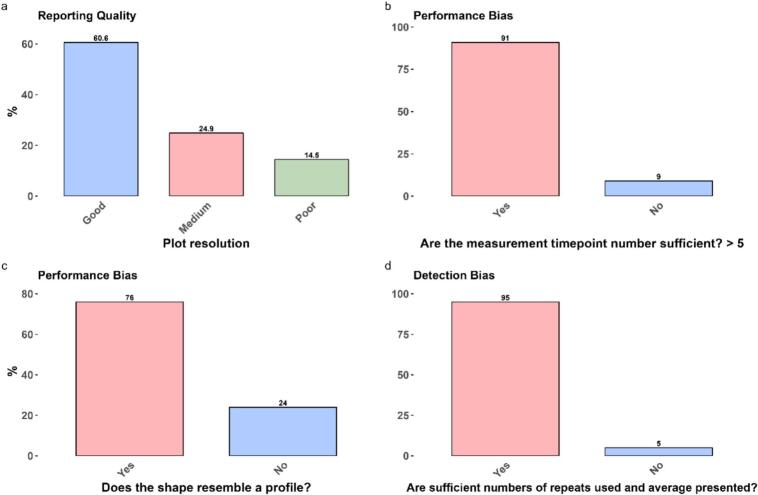
Assessing the quality of IVR plots found in articles returned using search method 1 and 2. IVR plots passed quality appraisal if they had a resolution of ‘good’ or ‘medium’ AND Yes across both performance bias detection bias metrics. A total of 221 / 271 IVR plots met these quality standards. The quality appraisal was conducted to evaluate the suitability of our digitised drug release data for AI-based drug release predictions.

Accurate and quantitative target output data is essential for accurate predictive AI models to predict drug release. To evaluate the suitability of our digitised IVR data for this purpose, we conducted a quality appraisal of the extracted drug release profiles. Variability in the number of data points per profile (Fig. S1) and in over data quality (Fig. 10) was observed. Of the 221 drug release profiles assessed, 159 met the quality appraisal criteria.

Currently, using IVR data from the literature as AI model outputs often requires manual extraction *via* software tools, which can introduce errors. To support AI-driven IVR predictions, we recommend that raw drug release data be provided in standardised CSV format, containing two columns: time and release %. It should be explicitly clear which formulation and testing conditions correspond to each IVR profile. Finally, all IVR data should meet our quality appraisal criteria (ESI section 1.5) to ensure data reliability and suitability for AI model development.

At present, there are minimum information reporting standards suggested in literature for nanoparticles in biological environments (Faria et al., 2018), but this does not focus on IVR tests. Based on this, a set of data standards are proposed to ensure nanomedicine formulation and IVR data is AI-ready.

### 3.4 Making IVR and formulation data AI-ready: Recommended data standards

The following data standards are essential for IVR, and formulation data generated to be used in AI-driven drug release predictions:

1. **Full formulation characterisation** of the nanomedicine product used for IVR studies is non-negotiable. As outlined in the MIRIBEL guidelines (Faria et al., 2018), at a minimum this includes particle size, polydispersity index (PDI), and zeta potential. With these inputs, AI practitioners can link formulation feature inputs to release kinetics.
2. **Precise formulation composition** reported as **molar percentages (mol %)** of each component of each nanomedicine product used for IVR studies is required. This format ensures accelerated integration into future versions of the database and could facilitate use of large language model data extraction.
3. **Complete IVR test method parameters** such as media volume, temperature, and pH must be reported. These are critical variables which affect release rate and can serve as feature inputs for AI-driven IVR predictions.
4. **High quality, machine-readable drug release data** must be supplied in raw format (*e.g.*, CSV with time and % release). Data must meet the quality appraisal criteria outlined above. Raw data enables both kinetic model fitting (Yanes et al., 2025) and/or direct input (Bannigan et al., 2023) approaches for AI-driven drug release predictions, while eliminating the need for error-prone extraction from plots.

## 4 Conclusion

This work presents a comprehensive, open-access database of liposome IVR experiments, along with data standards and a suggested database structure to standardise formulation and IVR data in nanomedicine. To our knowledge, this is the first nanomedicine database of this kind. This initiative was driven by current inconsistent reporting practices and a lack of databases suitable for AI-driven CQA and IVR predictions for liposomes. The database includes detailed information on formulation composition, preparation methods, and IVR testing conditions. It thus provides a foundation for generating larger, more diverse, and AI-ready datasets and for developing predictive AI models that can support nanomedicine formulation design and testing. By making the database open-access, it is intended to foster greater transparency and promote broader data sharing cultures within the pharmaceutics community. The proposed database structure and data standards are adaptable to other nanomedicine dosage forms, supporting wider efforts to harmonise data reporting in the field. Together, the database, structure, and standards aim to improve the traceability and utility of formulation and IVR data generated in nanomedicine research. Following these proposed standards will ensure data reported is AI-ready, enabling robust, predictive AI models to streamline nanomedicine research and fully harness the potential of AI in the field.

## CRediT authorship contribution statement

**Daniel Yanes:** Writing – review & editing, Writing – original draft, Visualization, Software, Methodology, Formal analysis, Data curation, Conceptualization. **Heather Mead:** Writing – review & editing, Supervision, Project administration. **James Mann:** Writing – review & editing, Writing – original draft, Supervision, Funding acquisition. **Magnus Röding:** Writing – review & editing, Writing – original draft, Supervision. **Vasiliki Paraskevopoulou:** Writing – review & editing, Supervision, Funding acquisition. **Cameron Alexander:** Writing – review & editing, Supervision, Funding acquisition. **Maryam Parhizkar:** Writing – review & editing, Writing – original draft, Supervision, Funding acquisition. **Jamie Twycross:** Writing – review & editing, Supervision, Funding acquisition. **Mischa Zelzer:** Writing – review & editing, Writing – original draft, Supervision, Project administration, Funding acquisition.

## Declaration of competing interest

M.R., V·P, J.M, H.M are employees of AstraZeneca and have stock ownership and/or stock options or interests in the company.

## Data Availability

The database, code, and datasets to generate the figures and reproduce the analysis are all accessible in the GitHub and Nottingham Research Data Management Repository, by using the following links: https://github.com/danielyanes22/nanomed_IVR_data.git and http://doi.org/10.17639/nott.7542

## Appendix A Supplementary data

The supplementary material contains additional methodological details on data extraction from the literature and the processing of literature data, examples for database entries to illustrate the database structure and additional data. Image 1
