# Supplementary material for: Making in vitro release and formulation data AI-ready: A foundation for streamlined nanomedicine development
Source: Int J Pharm X. 2025 Sep 11;10:100393. doi: 10.1016/j.ijpx.2025.100393 (PMC12481109; doi:10.1016/j.ijpx.2025.100393)
Supplement: Supplementary file 1 — The supplementary material contains additional methodological details on data extraction from the literature and the processing of literature data, examples for database entries to illustrate the database structure and additional data. [file mmc1.docx]

**Supplementary information**

**Making *in vitro* release and formulation data AI-ready: A foundation for streamlined nanomedicine development**

Daniel Yanes^1^, Heather Mead^2^, James Mann^2^, Magnus Röding^3,4^, Vasiliki Paraskevopoulou^2^, Cameron Alexander^1^, Maryam Parhizkar^5*^, Jamie Twycross^6^*, Mischa Zelzer^1*^,

^1^School of Pharmacy, University of Nottingham, University Park Campus, Nottingham, NG7 2RD, UK

^2^Global Product Development, Pharmaceutical Technology & Development, Operations, AstraZeneca, Macclesfield, SK10 2NA, UK

^3^Sustainable Innovation & Transformational Excellence, Pharmaceutical Technology & Development, Operations, AstraZeneca, Gothenburg, 43183 Mölndal, Sweden

^4^Department of Mathematical Sciences, Chalmers University of Technology and University of Gothenburg, 41296 Göteborg, Sweden

^5^School of Pharmacy, University College London, 29-39 Brunswick Square, London, WC1N 1AX, UK

^6^School of Computer Science, University of Nottingham, Jubilee Campus, Wollaton Road, Nottingham, NG8 1BB, UK

*Corresponding authors: maryam.parizkar@ucl.ac.uk; jamie.twycross@nottingham.ac.uk; mischa.zelzer@nottingham.ac.uk

# Supplementary methods

## Database construction and data acquisition

The methodology follows the same procedure we used previously (Yanes et al., 2025). For convenience and completion, we have reproduced the methodology section from the previous work (Yanes et al., 2025) below.

Three academic article searching methods were used to acquire data for entry into the database. Each article was manually assessed based on the criteria in **section: academic article inclusion criteria**.

### Article searching methods

Details of the specific search method, search engine, and resulting academic articles found are provided in the *search_terms* table in the *liposome_IVR.db* file. Whilst the details of the methodology used to find the article is provided below.

#### Method 1: Targeted articles for current FDA/EMA approved liposomes

Strings of search terms relating to currently approved Food and Drug Administration (FDA) and European Medicines Agency (EMA) liposomal products (Liu et al., 2022) were constructed (Table 1) and entered in either ScienceDirect or Google Scholar. Specifically, the search string included “Specific API name/product” AND either “*in vitro*” OR “drug” AND “release”. Results were filtered to include only research articles containing keywords which existed in either the article title or within the text.

Table S1: Current FDA/EMA approved liposomal products

| Product name | Active Pharmaceutical Ingredient |
| --- | --- |
| Doxil | Doxorubicin hydrochloride |
| Caelyx | Doxorubicin hydrochloride |
| DaunoXome | Daunorubicin |
| AmBisome | Amphotericin B |
| Myocet | Doxorubicin hydrochloride |
| Visudyne | Verteporfin |
| Mepact | Mifamurtide |
| Marqibo | Vincristine sulfate |
| Onivyde | Irinotecan hydrochloride trihydrate |
| Vyxeos | Daunorubicin |

#### Method 2: General liposome drug release

As there were limited articles that met our criteria found using method 1 due to confidentiality, the search was broadened. The above string was modified by changing “Specific API name/product” with “liposome” or “liposomal” and entered into Science Direct.

#### Method 3: Targeted string query

The string query below was constructed to target higher quality journals to improve the quality of reporting standards and to improve the search efficiency of the types of liposomes returned from the search. The query was entered into PubMed and the first 100 article sorted by best match returned were screened for suitability for data extraction based on criteria listed in **section: academic article inclusion criteria**.

("Nature nanotechnology"[Journal]) OR ("Journal of the American Chemical Society"[Journal]) OR ("J Control Release"[Journal]) OR ("Nat Commun"[Journal]) OR ("Nanomedicine"[Journal]) OR ("ACS nano"[Journal]) OR ("Nano letters"[Journal]) OR ("Advanced Functional Materials"[Journal]) OR ("Small"[Journal]) OR ("Biomaterials"[Journal]) OR ("Nano research"[Journal]) OR ("ACS Applied Materials & Interfaces"[Journal]) OR ("Nanoscale"[Journal]) OR ("Science advances"[Journal]) OR ("Science"[Journal]) OR ("Int J Pharm"[Journal]) OR ("Molecular Pharmaceutics"[Journal]) OR ("Eur J Pharm Biopharm"[Journal]) OR ("AAPS PharmSciTech"[Journal]) OR ("Acta Pharmacologica Sinica"[Journal]) OR ("Drug Delivery"[Journal]) OR ("Eur J Pharm Sci"[Journal]) OR ("Int J Nanomedicine"[Journal]) OR ("J Pharm Sci"[Journal]) AND ("liposomal" OR "liposome") AND ("In-vitro-release" OR "drug-release")NOT("Gel"[Title/Abstract])NOT (Review[Publication Type])NOT("ultrasound"[Title/Abstract])NOT("topical"[Title/Abstract])NOT("coated"[Title])NOT("nanocrystal"[Title/Abstract])NOT("polymer"[Title/Abstract])NOT("exosome"[Title/Abstract])NOT("siRNA"[Title/Abstract])NOT("mRNA"[Title/Abstract])NOT("hydrogel"[Title/Abstract])

### Academic article inclusion criteria

Each academic article was evaluated based on reported details of formulation type, formulation composition, formulation characterisation, drug release plot and IVR test methodology. Articles identified by the search engine to meet the search conditions were manually scanned for compliance with the additional criteria listed in the following sections.

#### Formulation type

Liposomal formulations which contained only one encapsulated API (no co-loading), small organic drug molecule encapsulation (not nucleic acids), conventional formulations (no solid lipid nanoparticles), and conventional compositions, e.g., no surface conjugation with antibodies.

#### Formulation composition

Full description of molar ratios of each component, or accessible information was required to be able to calculate molar ratios based on mass or accessible information of molar ratios on manufacturer websites. The description of mass of drug added to mass of excipients to calculate drug loading was also needed.

#### Formulation characterisation

Particle size measurements which used either dynamic light scattering or laser diffraction. If no characterisation data were available but a description of the specific formulation was given, the article was included as further preprocessing could be conducted. Encapsulation efficiency and experimental drug loading were not recorded due to the variability in methods used for this type of analysis.

#### Drug release plots

For inclusion, a plot must contain an identifier to the formulation that was used, supported with information relating to the formulation composition within the article. An article with a description of a formulation preparation but no details of the drug release test performed is rejected. The image size of the drug release plot must have been large enough to distinguish individual points, to allow accurate digitalisation. Axes must have contained drug release % or cumulative drug release %, data that plots concentration or amount of drug remaining is rejected.

#### IVR test methods

Niche applications of liposomal release were rejected (e.g., ultrasound probe attachments). Details of IVR test method type and media composition was required as a minimum, to ensure article selected could be used for both model fitting and a systematic review. It was accepted if the details of the individual components used to prepare the release medium were provided without specifying their individual concentrations. If the media pH and temperature were not reported, the article was accepted as further processing could be done.

### Database reporting

Articles varied in the level of detail reported for the description of IVR methods, e.g., amount of drug added, stirring (if required), aliquot volume, and post sampling treatment. Entries in the database were left as NULL if no details were reported. If post sampling treatment was left NULL in the database, it was assumed that either no details of post-sampling treatment was reported or no post-sampling treatment was required, i.e., a continuous flow methods such as modified USP-4 was used.

## Database structure

Table S2: Overview of database schema (structure) shown in Figure 2, including tables, columns, and descriptions for structured curation of formulation and *in vitro* release related literature data. Each table includes a primary key column named *ID* to ensure unique identification of each row and enabling referential integrity across relationships

| **Table** |  | **Field** | **Description** |
| --- | --- | --- | --- |
| Search_terms |  | Terms | Keywords of phrases used in the literature search |
|  |  | DB | Database where the terms were searched (e.g., PubMed, Scopus) |
|  |  | where_words_occur | Indicates where the search terms were found (e.g., title, abstract, full text) |
|  |  | Results | Number of search results returned |
|  |  | comments | Additional notes about the search |
|  |  |  |  |
| Papers |  | Title | Title of the article |
|  |  | Yr | Year the article was published |
|  |  | PDF | PDF file stored as binary large object (blob) |
|  |  | Num_formulations | Number of formulations reported in the paper |
|  |  | Search_ID | Foreign key linking to *search_terms* |
|  |  | Pdf_name | Name of the saved PDF |
|  |  | DOI | Digital object Identifier of the paper |
|  |  |  |  |
| API_name |  | API_name | Name of the active pharmaceutical ingredient (API) |
|  |  | SMILES | Simplified Molecular Input Line Entry System (SMILES) notation representing the API’s chemical structure |
|  |  |  |  |
| formulation |  | API_ID | Foreign key linking to *API_name* |
|  |  | Formulation_type | Type of formulation (e.g., liposome, nanoparticle) |
|  |  | Paper_ID | Foreign key linking to *papers* |
|  |  |  |  |
| Formulation_composition |  | Component_ID | Foreign key linking to *component_name* |
|  |  | Molar_ratio | Molar ratio of the component (excipient) in the formulation |
|  |  | Formulation_ID | Foreign key linking to *formulation* |
|  |  |  |  |
| Component_properties |  | Component_ID | Foreign key linking to *component_name* |
|  |  | Property_name | Name of the component property (e.g. molecular weight or literature phase transition temperature) |
|  |  | Property_value | Numerical value of the property |
|  |  |  |  |
| Component_name |  | Component_name | Name of the component (excipient) used in formulations |
|  |  |  |  |
| Formulation_CPPs_CQAs |  | Formulation_ID | Foreign key linking to *formulation* |
|  |  | Drug_loading | Amount of drug loaded into the formulation (w/w) |
|  |  | Preparation_method | Method used to prepare the formulation (e.g., Thin lipid film hydration and extrusion, microfluidics etc) |
|  |  | Encapsulation_method | Method used encapsulate the drug (e.g., ammonium sulfate gradient, simple mixing etc) |
|  |  | Incubation_temp_oC | Incubation temperature in °C |
|  |  | Incubation _time_hrs | Duration of incubation in hours |
|  |  | Comments | Additional remarks |
|  |  | PS_instrument | Instrument used for particle size analysis |
|  |  | Size_distribution | Description of type of size distribution used (e.g., volume, number etc) |
|  |  | Measurement_angle | Angle at which scattered light is detected relative to the incident laser beam (e.g., 15°, 90°, 175°) |
|  |  | Structure_type | Type of colloidal structure formed (e.g. multilamellar vesicle (MLV), small unilamellar vesicle (SUV), and large unilamellar vesicles (LMV). |
|  |  | Particle_size_nm | Measured particle size in nanometres |
|  |  | PDI | Polydispersity index |
|  |  | Zeta_potential | Zeta potential of the particles |
|  |  | Weighted_Tm | Experimental phase transition temperature in °C |
|  |  |  |  |
| IVR |  | Image_name | Name of image related to release profile |
|  |  | formulation_ID | Foreign key linking to *formulation* |
|  |  | formulation_name | Shorthand name for the formulation |
|  |  | Release_method | *In vitro* release method used (e.g., dialysis, USP-4 etc) |
|  |  | Drug_addition_vol_mL | Volume of drug solution added in mL |
|  |  | Stirring_rpm | Stirring speed in revolutions per minute |
|  |  | Aliquot_vol_mL | Volume of each aliquot taken at each time point in mL |
|  |  | Test_duration_Hrs | Total duration of the release study in hours |
|  |  | Dilution_media | Medium used to dilute aliquot |
|  |  | Dilution_factor | Factor by which the sample was diluted |
|  |  | Post_sampling_treatment | Treatment process after sampling (e.g. centrifugation, filtration etc) |
|  |  | Centrifuge_rpm | Speed of centrifugation in revolutions per minute |
|  |  | Centrifuge_time_min | Duration of centrifugation in minutes |
|  |  | Supernatatant_aliquout_uL | Volume of supernatant taken after processing in µL |
|  |  | Detection_method | Analytical method used to detect analyte (e.g., HPLC, UV) |
|  |  | Media_volume_mL | Volume of the release media in mL |
|  |  | Media_pH | pH of the media |
|  |  | Media_temp_oC | Temperature of the media in °C |
|  |  | Media_comp | Brief description of media composition |
|  |  | Time_units | Units for timepoints on graph e.g., minutes, hours |
|  |  |  |  |
| Media components |  | IVD_ID | Foreign key linking to *IVR* table |
|  |  | Component_name | Name of media component (e.g., Tween 90, NaCl etc) |
|  |  | Media_concentration_mM | Concentration of component in mM |
|  |  | Media_concentration_percent | Concentration of component as % |

## Digitalisation of literature data

To demonstrate the process associated with data extraction and entry, the following Tables S4 – S12 show the order in which the database tables should be populated for data entry and to illustrate the database structure for an example of data extraction from one academic article (Yuan et al., 2017). The primary key of the *IVR* table, i.e., the *ID* column, was used to name the digitised drug release profile. This primary key represents a unique identification link to the datatables for formulation, process, API, meta data, and IVR testing parameters.

Table S3: Data entry into the *search_terms* table for Yuan et al.

| ID | terms | db | where_words_occur | results |
| --- | --- | --- | --- | --- |
| 5 | doxil + liposomal + in vitro + drug release | SD | find articles with these terms | 394 |

Table S4: Data entry into the *papers* table for Yuan et al.

| ID | title | yr | pdf | num_formulations | search_ID | pdf_name | doi |
| --- | --- | --- | --- | --- | --- | --- | --- |
| 9 | Development of a flow-through USP-4 apparatus drug release assay to evaluate DLP | 2017 | *BLOB* | 5 | 5 | 1_9.pdf | 10.1208/s12248-016-9958-2 |

Table S5: Data entry into the *API_name* table for Yuan et al.

| ID | API_name | SMILES |
| --- | --- | --- |
| 1 | doxorubicin | COC1=CC=CC2=C1C(=O)C1=C(O)C3=C(C[C@](O)(C[C@@H]3O[C@H]3C[C@H](N)[C@H](O)[C@H](C)O3)C(=O)CO)C(O)=C1C2=O |

Table S6: Data entry into the *formulation* table for Yuan et al.

| ID | API_ID | formulation_type | paper_id |
| --- | --- | --- | --- |
| 21 | 1 | liposome | 9 |

Table S7: Data entry into the *component_name* table for Yuan et al.

| ID | component_name |
| --- | --- |
| 1 | HSPC |
| 2 | Cholesterol |
| 3 | mPEG2000-DSPE-2k |

Table S8: Data entry into the *formulation_composition* table for Yuan et al.

| ID | component_ID | molar_ratio | formulation_ID |
| --- | --- | --- | --- |
| 64 | 1 | 56.3 | 21 |
| 65 | 2 | 38.4 | 21 |
| 66 | 3 | 5.3 | 21 |

Table S9: Data entry into the *formulation_CPPs_CQAs* table for Yuan et al.

| ID | Formulation_ID | Drug_loading | Preparation_method | Encapsulation_method | Incubation_temp | incubation_time_Hrs | comments | … |
| --- | --- | --- | --- | --- | --- | --- | --- | --- |
| 21 | 21 | 0.125 | Lipid pouring and extrusion | Ammonium sulfate gradient | 60 | 1 | L-DOXp | … |

Table S10 (cont): Data entry into the *formulation_CPPs_CQAs* table for Yuan et al.

| PS_instrument | Size_distribution | Measurement_angle_0 | Structure_type | particle_size_nm | PDI | Zeta_potential | Weighted_Tm |
| --- | --- | --- | --- | --- | --- | --- | --- |
| DLS (Nano-ZS) | *NULL* | *NULL* | SUV | 91.0 | 0.042 | *NULL* | *NULL* |

Table S101: Data entry into the *IVR* table for Yuan et al.

| ID | image_name | formulation_ID | formulation_name | release_method | drug_addition_vol_mL | stirring_rpm | aliquot_vol_mL | test_duration_Hrs | … |
| --- | --- | --- | --- | --- | --- | --- | --- | --- | --- |
| 68 | 1_9_mediaconc | 21 | L-DOXp | ModifiedUSP-4 | 0.4 | *NULL* | *NULL* | 24.0 | … |
| 69 | 1_9_mediaconc | 21 | L-DOXp | ModifiedUSP-4 | 0.4 | *NULL* | *NULL* | 24.0 |  |
| 70 | 1_9_mediaconc | 21 | L-DOXp | ModifiedUSP-4 | 0.4 | *NULL* | *NULL* | 24.0 |  |
| 71 | 1_9_mediaconc | 21 | L-DOXp | ModifiedUSP-4 | 0.4 | *NULL* | *NULL* | 24.0 |  |
| 72 | 1_9_prep | 21 | L-DOXp | ModifiedUSP-4 | 0.4 | *NULL* | *NULL* | 24.0 |  |
| 76 | 1_9_MLV | 21 | L-DOXp | ModifiedUSP-4 | 0.4 | *NULL* | *NULL* | 24.0 |  |

Table S11 (cont): Data entry into the *IVR* table for Yuan et al.

| dilution_media | dilution_factor | post_sampling_treatment | centrifuge_rpm | centrifuge_time_min | supernatant_aliquot_uL | detection_method | … |
| --- | --- | --- | --- | --- | --- | --- | --- |
| direct | *NULL* | *NULL* | *NULL* | *NULL* | *NULL* | UV | … |
| direct | *NULL* | *NULL* | *NULL* | *NULL* | *NULL* | UV |  |
| direct | *NULL* | *NULL* | *NULL* | *NULL* | *NULL* | UV |  |
| direct | *NULL* | *NULL* | *NULL* | *NULL* | *NULL* | UV |  |
| direct | *NULL* | *NULL* | *NULL* | *NULL* | *NULL* | UV |  |
| direct | *NULL* | *NULL* | *NULL* | *NULL* | *NULL* | UV |  |

Table S11 (cont): Data entry into the *IVR* table for Yuan et al.

| media_volume_mL | media_pH | media_temp_oC | media_comp | Time_units |
| --- | --- | --- | --- | --- |
| 78.4 | 6.0 | 45.0 | 0 NH4HCO3 | hours |
| 78.4 | 6.0 | 45.0 | 25 NH4HCO3 | hours |
| 78.4 | 6.0 | 45.0 | 50 NH4HCO3 | hours |
| 78.4 | 6.0 | 45.0 | 100 NH4HCO3 | hours |
| 78.4 | 6.0 | 45.0 | HP-CD NH4HCO3 AMNS | hours |

Table S11: Data entry into the *media_components*  table for Yuan et al.

| ID | IVR_ID | component_name | media_concentration_mM | media_concentration_percent |
| --- | --- | --- | --- | --- |
| 129 | 68 | HP-CD | *NULL* | 5.0 |
| 130 | 68 | Ammonium bicarbonate | 0.0 | *NULL* |
| 131 | 68 | MES | 75.0 | *NULL* |
| 132 | 68 | NaN3 | *NULL* | 0.02 |
| 133 | 68 | sucrose | *NULL* | 5.0 |
| 134 | 69 | HP-CD | *NULL* | 5.0 |
| 135 | 69 | Ammonium bicarbonate | 25.0 | *NULL* |
| 136 | 69 | MES | 75.0 | *NULL* |
| 137 | 69 | NaN3 | *NULL* | 0.02 |
| 138 | 69 | sucrose | *NULL* | 5.0 |
| 139 | 70 | HP-CD | *NULL* | 5.0 |
| 140 | 70 | Ammonium bicarbonate | 50.0 | *NULL* |
| 141 | 70 | MES | 75.0 | *NULL* |
| 142 | 70 | NaN3 | *NULL* | 0.02 |
| 143 | 70 | sucrose | *NULL* | 5.0 |
| 144 | 71 | HP-CD | *NULL* | 5.0 |
| 145 | 71 | Ammonium bicarbonate | 100.0 | *NULL* |
| 146 | 71 | MES | 75.0 | *NULL* |
| 147 | 71 | NaN3 | *NULL* | 0.02 |
| 148 | 71 | sucrose | *NULL* | 5.0 |
| 184 | 72 | HP-CD | *NULL* | 5.0 |
| 185 | 72 | Ammonium bicarbonate | 100.0 | *NULL* |
| 186 | 72 | MES | 75.0 | *NULL* |
| 187 | 72 | NaN3 | *NULL* | 0.02 |
| 188 | 72 | sucrose | *NULL* | 5.0 |
| 204 | 76 | HP-CD | *NULL* | 5.0 |
| 205 | 76 | Ammonium bicarbonate | 100.0 | *NULL* |
| 206 | 76 | MES | 75.0 | *NULL* |
| 207 | 76 | NaN3 | *NULL* | 0.02 |
| 208 | 76 | sucrose | *NULL* | 5.0 |

## DATASET CONSTRUCTION

Structured Query Language (SQL) queries were constructed to join *formulation_CPPs_CQAs* and *IVR* tables into a singular dataframe. Then, the calculated *API_properties* were joined onto the resultant dataset creating a singular dataset. This new dataset was probed to assess qualitative relationships between drug, formulation properties, and IVR method parameters.

## Quality appraisal of drug release plots

A quality appraisal of each of the digitised drug release plots was conducted to ensure that the plots were suitable for kinetic model fitting. This consisted of an assessment of reporting quality, performance, and detection bias.

The reporting quality was assessed on the basis of plot resolution: ‘good’, ‘medium’ or ‘poor’. ‘Good’ plots consisted of clear and distinguishable individual data points, ‘Medium’ plots had reasonably distinguishable data points and ‘Poor’ plots had overlapping points with other release profiles and/or had a small datapoint size. Performance bias was assessed via the following questions: ‘Are measurement timepoint numbers sufficient, i.e., greater than 5?’ and ‘Does the curve shape resemble a profile that could be subjected to kinetic model fitting?’. Detection bias consisted of ‘Is the number of repeats equal or larger than 3 and is the average of the repeat measurements presented?’. For a profile to pass the quality appraisal it would have to have a resolution of ‘good’ or ‘medium’ AND score ‘yes’ across both performance bias metrics.

# Supplementary figures


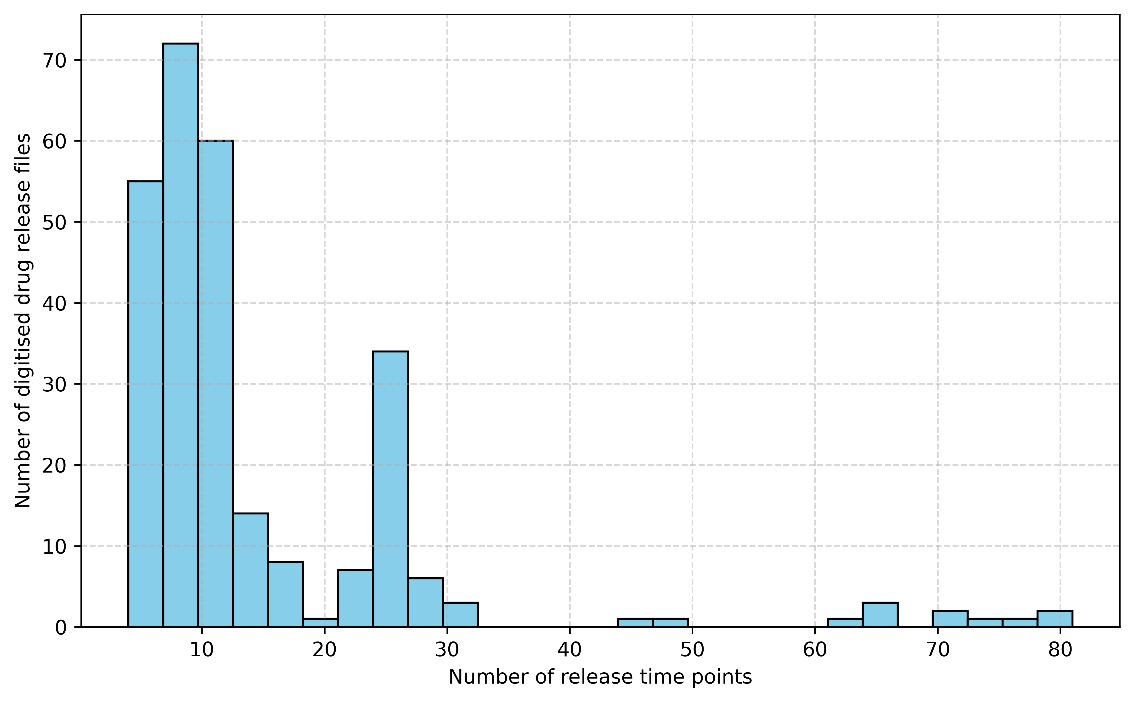


Figure S1: Number of data points in each extracted drug release file. Black diamonds represent outliers, determined as datapoints that fall outside the 1.5 x interquartile range.

# Supplementary tables

Table S13: Drug molecular descriptors used. Molecular descriptors were calculated using RDKit.

| Compound | MolWt | TPSA | Num  HAcceptors | Num  HDonors | Num  Rotatable  Bonds | MolLogP |
| --- | --- | --- | --- | --- | --- | --- |
| doxorubicin | 543.53 | 206.07 | 12 | 6 | 5 | 0 |
| amphotericin B | 924.09 | 319.61 | 17 | 12 | 3 | 0.71 |
| methotrexate | 454.45 | 210.54 | 10 | 5 | 9 | 0.27 |
| carboxyfluorescein | 378.34 | 121.13 | 5 | 3 | 3 | 2.89 |
| propranolol | 259.35 | 41.49 | 3 | 2 | 6 | 2.58 |
| atenolol | 266.34 | 84.58 | 4 | 3 | 8 | 0.45 |
| budesonide | 430.54 | 93.06 | 6 | 2 | 4 | 2.72 |
| hydrocortisone | 362.47 | 94.83 | 5 | 3 | 2 | 1.78 |
| bupivacaine | 288.44 | 32.34 | 2 | 1 | 5 | 3.9 |
| rhodamine b | 479.02 | 56.69 | 3 | 1 | 7 | 2.57 |
| vincristine sulfate | 923.05 | 245.77 | 14 | 5 | 8 | 2.86 |
| amoxicillin | 365.41 | 132.96 | 6 | 4 | 4 | 0.02 |
| calcein | 622.54 | 231.67 | 11 | 6 | 12 | 1.61 |
| paclitaxel | 853.92 | 221.29 | 14 | 4 | 10 | 3.74 |
| gemcitabine | 263.2 | 110.6 | 7 | 3 | 2 | -1.29 |
| 5-Fluorouracil carbonate | 400.49 | 90.39 | 6 | 1 | 15 | 4.49 |
| Hydroxy-alpha-sanshool | 263.38 | 49.33 | 2 | 2 | 8 | 2.9 |
| BAY 87-2243 | 525.54 | 85.34 | 9 | 0 | 7 | 4.53 |
| fraxetin | 208.17 | 79.9 | 5 | 2 | 1 | 1.21 |
| temoporfin | 680.76 | 138.28 | 6 | 6 | 4 | 9.76 |
| sulforhodamine B | 558.68 | 130.96 | 7 | 1 | 8 | 4.01 |
| tetrodotoxin | 319.27 | 190.25 | 11 | 8 | 1 | -5.52 |

# references

Liu, P., Chen, G., Zhang, J., 2022. A Review of Liposomes as a Drug Delivery System: Current Status of Approved Products, Regulatory Environments, and Future Perspectives. Molecules 27, 1372. https://doi.org/10.3390/molecules27041372

Yanes, D., Paraskevopoulou, V., Mead, H., Mann, J., Röding, M., Parhizkar, M., Alexander, C., Twycross, J., Zelzer, M., 2025. to accelerate the design of in vitro release tests from liposomes. https://doi.org/10.17639/nott.7522

Yuan, W., Kuai, R., Dai, Z., Yuan, Y., Zheng, N., Jiang, W., Noble, C., Hayes, M., Szoka, F.C., Schwendeman, A., 2017. Development of a Flow-Through USP-4 Apparatus Drug Release Assay to Evaluate Doxorubicin Liposomes. AAPS J. 19, 150–160. https://doi.org/10.1208/s12248-016-9958-2
